# Are repetitive pericranial nerve blocks effective in the management of chronic paroxysmal hemicrania? A case report

**DOI:** 10.1097/MD.0000000000016484

**Published:** 2019-08-02

**Authors:** Devrimsel Harika Ertem

**Affiliations:** Department of Neurology, Sisli Hamidiye Etfal Training and Research Hospital, University of Health Sciences, Istanbul, Turkey.

**Keywords:** greater occipital nerve block, headache, pain, pain management, paroxysmal hemicrania, sphenopalatine ganglion block, trigeminal convergence

## Abstract

**Introduction:**

Paroxysmal hemicrania (PH) is a chronic headache disorder characterized by unilateral pain attacks accompanied by cranial autonomic symptoms and responds to indomethacin completely. There are few alternative treatment options for the patients who cannot tolerate indomethacin. Studies exploring the effects of repetitive peripheral cranial nerve blocks in the management of chronic PH are limited.

**Patient concerns and diagnosis:**

A 34-year-old woman with a 4-year history of PH was evaluated. Her pain was prevented by indomethacin without side effects; however, she wanted to try to conceive.

**Interventions:**

Repetitive pericranial nerve blocks, great occipital nerve, infraorbital nerve, supraorbital nerve, and sphenopalatine ganglion block, using local anesthetics and steroids were performed once a week for a 6 weeks period.

**Outcomes:**

A follow-up of 3 months showed that there was no pain relief following the injections and patient was needed to be maintained on indomethacin.

**Conclusion:**

Although pericranial nerve blocks have been tried in chronic PH cases with positive influences, this case indicated that repetitive nerve blocks were not always a successful therapy option.

## 1 Introduction

According to the International Classification for Headache Disorders (ICHD-3), the clinical features of paroxysmal hemicrania (PH) include recurrent severe and unilateral headache attacks, lasting 2 to 30 minutes with ipsilateral autonomic features such as conjunctival injection, lacrimation, nasal congestion, rhinorrhea, sweating, miosis, and/or ptosis. This type of trigeminal autonomic headache responds absolutely to indomethacin treatment.^[1]^ Topiramate, verapamil, lithium, carbamazepine, gabapentin, or transcutaneous vagus nerve stimulation may be used in treatment where indomethacin side effects are seen or contraindicated.^[2]^ As PH is a relatively rare type of headache, there are no oral medication clinical trials when indomethacin treatment fails. Randomized and placebo-controlled clinical trials have reported that occipital nerve and sphenopalatine ganglion blocks (SPGBs) are effective interventional approaches to treat primary headache disorders such as migraine, cluster headache, and chronic daily headache.^[3,4]^ The efficacy of peripheral nerve blocks in the treatment of PH are mostly derived from case reports and experience. In this case report, it was aimed to present the experience of interventional pain management in an adult woman who had repetitive peripheral nerve blocks in the treatment of chronic PH as an alternative to oral medication and review of the current literature data.

## 2 Case report

A 34-year-old woman with a diagnosis of PH for 4 years was referred to headache clinic for preconception counseling. She had about 7 to 15 headache episodes per day, and her headaches are characterized by a unilateral, moderate to severe, occasionally nausea, and photophobia, localized to left orbital, supraorbital, and occipital areas, sharp and throbbing pain radiating to same side neck and shoulder, accompanied by ipsilateral autonomic features such as eyelid edema, ptosis, lacrimation, and nasal congestion and/or rhinorrhea lasting 5 to 15 minutes. Those headaches typically responded well to indomethacin. She was being treated with indomethacin 25 mg 3 times a day and lansoprazole 15 mg once a day (proton pump inhibitor). The numeric rating scale (NRS) score was 8 to 9 out of 10. She did not have a history of headache trauma and nocturnal headaches. She reported that when she stopped using indomethacin, her headache attacks recurred. She had normal neurological examination, blood, urine, and coagulation tests. She complained of some tenderness to palpation over the left temple, occipital and neck sites, and regional pain sensitivity was described by noxious pressure. Contrast enhanced magnetic resonance (MR) imaging of the brain and MR angiography were both normal. The patient met the ICHD-3 diagnostic criteria for chronic PH. She reported that she wanted to try to conceive within the next few months. Interventional pain management by performing repetitive peripheral cranial nerve and SPG blocks was planned. Informed consent for publication has been obtained from the patient.

### 2.1 Injection procedure

The patient gave her consent for interventional pain treatment. Change of pain severity in the NRS was used to assess the response to nerve blocks. The patient stopped taking indomethacin during the procedures. At first, a mixture of local anesthetic 4 mL of 2% lidocaine and 1 mL of methylprednisolone acetate was injected at the left great occipital nerve (GON) located at approximately 10 mm medial to the midpoint of the line of the occipital tubercle and the mastoid tip. Mild pain relief effect had been presented within the first week. In addition to GON block, left infraorbital and supraorbital nerve (SON) blocks with 1 mL of 2% lidocaine were performed once a week for 2 weeks. However, the number of attacks and pain severity were not improved after 3 weeks. SPGB was performed with sterile cotton sticks, soaked in 10% lidocaine, inserted through the patient's nose to the back of nasopharynx for 30 minutes, once a week for 3 weeks. Pain frequency and severity were not significantly changed. The degree of pain remained at NRS 8 to 9 at 6 weeks after the first injection. She was continued on treatment with indomethacin. No adverse effects of the procedures were reported.

A follow-up of 3 months showed that there was no pain relief following the injections and patient was needed to be maintained on indomethacin.

## 3 Discussion

In the present study, a negative response to GON, SON, infraorbital nerve, and SPG blocks in managing chronic PH in a young adult woman with a diagnosis of chronic PH was demonstrated. Data concerning adult patients with PH refractory to conventional treatment are scarce. Indomethacin is a nonsteroidal anti-inflammatory drug with potent analgesic and anti-inflammatory activity used as acute and preventative treatment for PH headache since the 1960s.^[5]^ Furthermore, the diagnosis of PH is clinically supported by a response to indomethacin.^[1]^ Even though indomethacin is inexpensive and easy to administer, the side effects associated with indomethacin such as gastrointestinal bleeding, coagulation disorders, renal dysfunction, and hypertension may limit its use.^[6]^ The patient in the current report had full recovery from headache attacks by using indomethacin. She was referred to headache clinic for a preconception counseling consultation. Indomethacin is labeled as category C in pregnancy by the US Food and Drug Administration (FDA), and there are animal studies which show an adverse effect on the fetus and there are no adequate and well-controlled studies in humans. Therefore, preconception counseling is crucial for patients who are under indomethacin treatment before pregnancy to reduce chances of poor perinatal outcomes.^[7]^ Several previous studies reported that peripheral nerve blocks on GON, SON, infraorbital nerves, or SPG are effective in controlling PH headaches.^[8,9]^ Due to the fact that this type of headache is rare, most literature on PH has been limited to case reports or case series, involving patients who are intolerant to therapeutic doses of indomethacin and resistant to multiple medication.

In current literature, there are limited data evaluating the effects of pregnancy on PH. In a study of Boes et al,^[10]^ the authors explored the clinical spectrum of 74 patients with chronic PH. They reported that 1 of these patients stopped indomethacin as she was trying to conceive. She had daily PH headaches in the first trimester; however, she did not have pain in her second and third trimesters. The purpose of interventional pain management for the patient presented here is to reduce pain intensity and frequency via repetitive cranial nerve blocks to overcome the need for indomethacin during pregnancy.

Although research on the efficacy of peripheral nerve blocks in the treatment of migraine and cluster type headaches indicate that cranial nerve blocks might be an alternative treatment for refractory headaches, the effects of GON, SON, infraorbital, and SPG blocks in PH cases still remain unclear. The GON is a branch of the second cervical nerve and there are convergent inputs between the caudal trigeminal nucleus and upper cervical segments leading to cranial nociception.^[11]^ Due to trigeminovascular autonomic reflex by hypothalamo-trigeminal connections, cranial autonomic symptoms are prominent in PH. Infiltrating the SPG may block the nerve connections from hypothalamic centers to the SPG. Due to local anesthetics and corticosteroids block the transmission in the nociceptive nerves, it has been hypothesized that blocking this activity of cranial nerves and SPG may be helpful in the treatment of PH. Successful GON, SON, infraorbital, and SPG blocks with anesthetics with or without corticosteroids in patients with PH have been reported previously.^[8,9]^ However, in the current report, repetitive peripheral nerve blocks were not effective in managing PH. Antonaci et al^[8]^ reported anesthetic blocks of cranial nerves of 6 patients with chronic PH. They noted that blocking symptomatic side in patients with chronic PH by lidocaine responded negatively. However, SON blocks reduced the pain intensity significantly. In a brief report of Morelli et al,^[9]^ the authors reported a 69-year-old woman suffering from PH and resistant to multiple therapies. They performed endoscopic SPG block approaching the pterygopalatine fossa and injected a mixture of local anesthetics bupivacaine and mepivacaine with and steroids. They observed that patient's clinical features were dramatically modified following the SP endoscopic ganglion block and they proposed that SPG blockade could be considered a reasonable alternative for drug-resistant PH patients. After the procedures, complaints of the patient of the current report did not disappear acutely and a follow-up of 3 months showed that there was no clinical improvement. Different responses to cranial nerve and SPG blocks may be explained by involving additional and multifactorial aspects.

Chronic PH is a rare disorder, and there is no recognizable scheme for treatment where indomethacin is contraindicated yet. Although peripheral nerve and SPG blocks could be considered as a reasonable option in drug-resistant PH cases due to abovementioned anatomical and physiological functions and processes, this case report indicated that blocks on GON and SPG resulted in ineffective for controlling chronic PH. Further studies are needed to explore the effects of the nerve blocks for the treatment of PH headaches.

## Author contributions

**Conceptualization:** Devrimsel Harika Ertem.

**Data curation:** Devrimsel Harika Ertem.

**Methodology:** Devrimsel Harika Ertem.

**Resources:** Devrimsel Harika Ertem.

**Writing – original draft:** Devrimsel Harika Ertem.

**Writing – review & editing:** Devrimsel Harika Ertem.

Devrimsel Harika Ertem orcid: 0000-0001-5309-1258.

Devrimsel Harika Ertem orcid: 0000-0001-5309-1258.
